# Current approach in the diagnosis and management of panuveitis

**DOI:** 10.4103/0301-4738.58471

**Published:** 2010

**Authors:** Reema Bansal, Vishali Gupta, Amod Gupta

**Affiliations:** Department of Ophthalmology, Advanced Eye Centre, Post Graduate Institute of Medical Education and Research, Chandigarh, India

**Keywords:** Diagnosis, management, panuveitis

## Abstract

Panuveitis is a generalized inflammation of not only the whole of the uveal tract but also involves the retina and vitreous humor. It differs from other anatomical sites of inflammation in terms of causes as well as distribution. The common causes of panuveitis in our population are tuberculosis, Vogt-Koyanagi-Harada syndrome, sympathetic ophthalmia, Behcet's disease and sarcoidosis. A large number of cases still remain idiopathic. A stepwise approach is essential while evaluating these patients to be able to identify and treat the disease timely and correctly. Ancillary tests can be appropriately applied once the anatomic site of inflammation is identified. An exhaustive approach comprising a full battery of tests is obsolete. Only specific tailored investigations are ordered as suggested by the preliminary clinical and ocular examination. The mainstay of the treatment of uveitis is corticosteroids. Immunosuppressive agents are administered if the inflammation is not adequately controlled with corticosteroids. One of the recent breakthroughs in the treatment of refractory uveitis includes the introduction of immunomodulating drugs: Tumor necrosis factor-alpha antagonist and Interferon-alpha. Vitrectomy has been used in uveitis for over a few decades for diagnostic and therapeutic purposes. When compared to other anatomical sites of inflammation, panuveitis has poor visual outcome due to more widespread inflammation. The side-effects of the chronic treatment that these patients receive cannot be overlooked and should be specifically monitored under the supervision of an internist with special interest in inflammatory diseases.

Uveitis is one of the major causes of blindness in the world.[1] According to anatomical location, the International Uveitis Study Group (IUSG) defines panuveitis as generalized inflammation of all three parts of the uvea, i.e., iris, ciliary body and the choroid.[2] It covers a large group of diverse diseases, which affect not only the uvea but also the retina and vitreous humor. Diagnosis of panuveitis is established in the presence of the following clinical signs:

- Evidence of choroidal or retinal inflammation such as choroiditis (focal, multifocal or serpiginous), choroidal granuloma, retinochoroiditis, retinal vasculitis, subretinal abscess, necrotizing retinitis or neuroretinitis; with
- evidence of vitreous inflammation (vitreous cells or vitritis); and
- presence of signs of anterior uveitis (cells and flare in the anterior chamber, keratic precipitates or posterior synechiae).

The distribution of uveitis according to the anatomical site of inflammation and its causes are influenced by diverse geographic, racial, nutritional and socioeconomic differences.[3] Knowledge of the epidemiology of uveitis helps the clinician to better predict the likelihood of a systemic association and to order appropriate diagnostic testing.[4] Panuveitis is relatively more common in Asia, Africa and South America as compared to North America, Europe and Australia.[5] Tuberculosis (TB) and Vogt-Koyanagi-Harada (VKH) syndrome are the most common causes of panuveitis in India.[3,6]

This article highlights the current clinical approach to the diagnosis and management of panuveitis, with special emphasis on the most common causes of panuveitis in our setting.

## Clinical approach

Classification and standardization of uveitis is important, as it enhances the precision and comparability of clinical examination. This helps in developing a complete picture of the course of the disorders and their response to treatment. The most widely used classification of uveitis is the one devised by the IUSG in 1987, based on anatomical location of inflammation. The standardization of uveitis nomenclature working group in 2005 standardized the approach to reporting clinical data (diagnostic terminology, grading of inflammation and outcome measures) in uveitis research.[7] More recently, the IUSG in 2008 designed a simplified classification of uveitis into infectious, non-infectious and masquerade, based on etiological criteria.[8]

To recognize a specific entity, we use the following step-ladder approach when evaluating a patient with uveitis:

1. A detailed ocular history is elicited regarding the symptoms, duration (acute or chronic), number of episodes (recurrent), and laterality of the symptoms.
2. Thorough ocular examination is done to assess the anatomic location of the uveitis, type of inflammation (granulomatous or non-granulomatous), to recognize any typical entity (such as Fuchs' heterochromic cyclitis).
3. Once the naming and meshing of the disease is done, systemic history (targeted questions) is asked to know any associated conditions or syndromes causing uveitis.
4. Only selective investigations are ordered.

## Investigations

Nearly all cases of uveitis require investigations for correct diagnosis (or label) and guidance for treatable conditions (infections), to know the natural history and prognosis of the disease, to provide reassurance to the patient as well as doctor, to identify groups at particular risk [antinuclear antibody (ANA) status in juvenile idiopathic uveitis] and to avoid unnecessary treatments. Clinically, uveitis can be classified into granulomatous and non-granulomatous uveitis. Anatomic approach is essential in the diagnosis of uveitis for proper application of ancillary tests.

### Ancillary tests

Whereas anterior segment inflammations involving the iris are routinely picked up by slit-lamp examination, ciliary body involvement can best be confirmed by ultrasound biomicroscopy (UBM). Baseline color fundus photography serves as an extremely good clinical reference when monitoring the fundus changes during follow-up periods. Digital color fundus photography allows easy storage and retrieval of fundus images. A composite montage of the retina can be made to document better the lesions in the peripheral fundus.[9]

Fundus fluorescein angiography (FA) is mandatory for retinal and choroidal lesions. The FA allows identification of active inflammation of the retinal vessels as seen in vasculitis due to sarcoidosis, tuberculosis, Behcet's disease and syphilis. The pattern of staining and leakage provides diagnostic clues. Vascular occlusions can also be detected. Clinically absent macular edema or optic disc edema can be revealed on FA. Besides the active lesions, complications of uveitis that are best studied on FA include cystoid macular edema (CME), neovascularization of retina, capillary dropout, subretinal neovascularization and retinal pigment epithelium (RPE) changes. Exudative retinal detachment in the early stage of VKH begins as bilateral, multifocal points in the posterior pole. Multifocal leaks at the level of RPE with pooling of the dye in the areas of exudative detachment are typically seen on the FA in VKH patients. Similar lesions on FA may also be seen in sympathetic ophthalmia. Staining of a hypofluorescent lesion starting at the border and then progressing to the center is typical of toxoplasmic retinochoroiditis. Indocyanine green angiography (ICGA) is the technique of choice for imaging the choroid. The alteration of the normal choroidal ICGA background fluorescence is the main parameter studied, and information is obtained mostly from late angiographic phases when choroidal inflammatory lesions may appear as areas of decreased or absent fluorescence. Using ICGA, choroidal vasculitis in posterior uveitis can be characterized and subdivided into two main patterns: (1) primary inflammatory choriocapillaropathy and (2) stromal inflammatory vasculopathy.[10] The first pattern consists of hypofluorescent areas up to the late phase of angiography characteristic of choriocapillaris non-perfusion and includes entities such as multiple evanescent white dot syndrome, acute posterior multifocal placoid pigment epitheliopathy, multifocal choroiditis, ampiginous choroiditis and serpiginous choroiditis. The second pattern consists of fuzzy indistinct appearance of vessels in the intermediate angiographic phase and diffuse choroidal hyperfluorescence in the late phase indicating inflammatory vasculopathy of larger choroidal vessels. This pattern has been described in active VKH disease, ocular sarcoidosis and tuberculosis and birdshot chorioretinopathy. In Behçet's uveitis of recent onset, choriocapillaris perfusion delay and fuzzy choroidal vessels without diffuse late choroidal hyperfluorescence has been described. Ultrasound is a safe, noninvasive, dynamic tool for the evaluation of the posterior segment when direct visualization of the fundus is obscured due to severe inflammation or its complications. It is useful also for the evaluation of inflammatory infiltration of the choroids in VKH syndrome or sympathetic ophthalmia (SO).[11] It is especially helpful in differentiating choroidal thickening associated with choroiditis from posterior scleritis which may mimic posterior or even panuveitis. It is also useful when evaluating patients prior to the use of intraocular drugs or surgery. UBM allows an objective, quantitative evaluation of the ciliary body.[12] It can be of great help in deciding the course of treatment by detecting the underlying structural abnormalities in ocular hypotony associated with uveitis.[13]

Optical coherence tomography (OCT) is a noncontact and noninvasive imaging tool. Although certain complications of uveitis such as CME, neovascularization of retina, epiretinal membrane, or vitreomacular traction syndrome can be demonstrated extremely well on the OCT, its use in panuveitis may be restricted by hazy media. Recently, our experience with the use of OCT in uveitic eyes has revealed that high-definition (HD) spectral-domain OCT [SD-OCT] (Cirrus HD-OCT; Carl Zeiss, Dublin, California, USA) has an advantage over time-domain OCT [TDOCT] (Stratus version 4; Carl Zeiss) for imaging macula in patients of uveitis by providing better identification of normal and pathologic structure in patients with poor media clarity.[14]

### Laboratory tests

Recent advances in the understanding of the pathogenetic mechanisms of uveitis have changed the diagnostic and therapeutic approach to these patients. In most textbooks, the exhaustive approach is presented. Such lists are not very useful. Random screening with a full battery of tests is needless. One should concentrate on a limited number suggested by naming-meshing. We routinely order the following laboratory tests:

- Full blood counts
- Erythrocyte sedimentation rate
- Mantoux test
- Chest X-ray (Computed tomography if required)
- Syphilis serology (*Treponema pallidum* hemagglutination test)
  The following tests are ordered only in relevance to the particular disorder:
- Serum Angiotensin-Converting-Enzyme (ACE) levels for sarcoidosis
- Human leukocyte antigen (HLA) typing (B 51, DR4) for Behcet's disease (BD) or VKH syndrome
  (Although BD is associated with the HLA-B51 locus, not all patients have this genotype[15])
- ANA for juvenile rheumatoid arthritis and antineutrophil cytoplasmic antibody for vasculitis associated with Wegener granulomatosis
- X-ray of sacro-iliac joint for ankylosing spondylitis
- Antibodies against *Toxoplasma gondii*

A repeat evaluation and follow-up is required in cases where laboratory test results do not yield any positive information. Cases that remain undiagnosed by the routinely available laboratory tests are labeled as idiopathic.[3]

In some of the etiologies (infectious endophthalmitis, acute retinal necrosis), a clinical diagnosis can be made and therapy can be immediately started, while the specific confirmation tests are being ordered or carried out. Whereas culture still remains the gold standard for diagnosing microbial infections, definitive diagnosis of intraocular inflammation due to infectious agents is difficult to obtain from ocular fluids or tissue specimens in routine clinical practice. Our ability to detect infectious agents has been strengthened by the use of polymerase chain reaction (PCR). It has been used to diagnose uveitis, including viral uveitis, mycobacterial intraocular infections, infectious endophthalmitis, and protozoal eye diseases.[16] PCR is a powerful molecular technique for evaluation of very small amounts of Deoxyribonucleic acid and Ribonucleic acid. It can be a simple, rapid, sensitive, and specific tool for the diagnosis of infection, autoimmunity, and masquerade syndromes in the eye.

Certain tests such as liver function and renal function tests are ordered only during the course of treatment while evaluating a patient before considering immunosuppressive therapy or while evaluating the adverse effects of these drugs or certain antimicrobial drugs.

## Management of Panuveitis

The treatment of uveitis has three main goals: to prevent vision-threatening complications, to relieve the patient's complaints and, when feasible, to treat the underlying disease.[17] It can be divided into following steps:

- Diagnosis and treatment of the specific causative agent.
- Nonspecific treatment.
- Treatment of related conditions.
- Supportive therapy.

Uveitis due to infectious agents is treated by specific antimicrobial therapy (antibiotic, antiparasitic or an antiviral) for appropriate duration, with or without corticosteroids. The mainstay of treatment of noninfectious uveitis is anti-inflammatory therapy. Severe or refractory panuveitis needs immuno-suppressive agents.

### 

#### Corticosteroids

Corticosteroids are the drugs of choice in most types of uveitis. They inhibit the inflammatory process by suppressing the arachidonic acid metabolism and activation of complement.[17]

In panuveitis, both topical and systemic corticosteroids are needed. Depending upon the severity of the disease, oral prednisolone is started in a loading dose of 1 mg/kg/day. As the inflammation subsides, tapering of corticosteroids by 5-10 mg per week is begun within two to four weeks of initiating therapy. Once the eye is completely quiescent, the patient is followed on a maintenance dose ranging from 2.5-10 mg daily of prednisolone. A reasonably long period of low-dose corticosteroids is required as maintenance therapy in VKH syndrome and SO.

The normal response to the corticosteroid therapy may be interrupted by recurrence of uveitis in which case the frequency of instillation of topical drops is increased besides raising the oral corticosteroid to the initial high-dose levels. Unilateral cases may be given a trial with periocular injection of depot corticosteroids into the posterior subtenon space. The side-effects and complications of topical or systemic corticosteroids must be looked for at every follow-up visit of the patient. These include secondary glaucoma, posterior subcapsular cataract, increased susceptibility to infection (ocular or systemic), hypertension, gastric ulcer, diabetes, obesity, growth retardation, osteoporosis and psychosis.

#### Supportive therapy

Cycloplegics are administered to relieve pain due to ciliary spasm. Posterior synechiae formation is prevented by instilling a mydriatic agent. Atropine is used in acute attacks while intermediate acting agents (homatropine) are used to maintain pupillary dilatation.

#### Immunosuppressive agents

The three main classes of immunosuppressives that are widely used today in addition to glucocorticosteroids are antimetabolites, T cell inhibitors and alkylating agents. Antimetabolites include azathioprine, methotrexate and mycophenolate mofetil (MMF). T cell inhibitors include cyclosporine and tacrolimus. Alkylating agents include cyclophosphamide and chlorambucil. When corticosteroid therapy is insufficient to control ocular inflammatory disease, immunosuppressive agents are given. They exert their beneficial effects by actually killing the rapidly dividing clones of lymphocytes that cause the inflammation. Indications of immunosuppressive therapy in panuveitis are: severe inflammation that is sight-threatening; chronic inflammation that is not responding to the primary conventional corticosteroid therapy; multiple relapses of uveitis; or intolerance or contraindications to systemic corticosteroids. The clinician should discuss extensively with the patient regarding the side-effects of such therapy.

These drugs have to be used only after ruling out a possible infectious agent as the cause of uveitis. VKH syndrome and SO are the uveitis conditions that are usually resistant to corticosteroids or require long-term treatment with corticosteroids. In such conditions, immunosuppressive agents are initiated as immediate second line therapy or as steroid-sparing agents as first-line therapy. All patients are evaluated for hemoglobin, blood cell counts (leucocytes and platelets), liver and renal function tests to rule out contraindications to treatment before starting any immunosuppressive drug, and every four weeks while receiving these medications. Low-dose immunosuppressive drugs such as azathioprine or methotrexate (MTX) are also initiated before any intraocular surgery to control the inflammation for a prolonged time and sustain it after surgery for a favorable outcome.[18,19]

Cyclosporine[20] and azathioprine[21] have been found to be effective in the treatment of Behcet's disease in randomized controlled trials, whereas the efficacy of other agents is shown by uncontrolled case series.

#### Newer strategies

Biologic drugs were introduced as an alternative mode of therapy for recalcitrant uveitis about 15 years ago, with encouraging outcomes. These are therapeutic agents with biologic properties, including monoclonal antibodies and soluble cytokine receptors. The main biologics in current use include anti-tumor necrosis factor-α (TNF-α), cytokine receptor antibodies and interferon-α (IFN-α). These are believed to have a superior anti-inflammatory potential than the conventional immunosuppressives and have been proposed as a second-line strategy after failure with conventional immunosuppressants for the treatment of refractory uveitis, especially with ocular BD.[22]

### Anti-tumor necrosis factor-α

TNF-α is an inflammatory cytokine found in animal models of uveitis as well as the aqueous of eyes with uveitis. The three currently commercially available anti-TNF-α agents are infliximab, adalimumab and etanercept. Infliximab and adalimumab are monoclonal immunoglobulin G1 (IgG1) antibodies against TNF-α. They both form stable bonds with the soluble and transmembrane forms of TNF-α. Etanercept is a dimeric soluble form of the p75 TNF-α receptor linked to IgG1 and forms less stable bonds mainly with the transmembrane form. Anti-TNF-α agents are increasingly proving to be effective in the control of uveitis. Infliximab in particular has been found to be effective in reducing inflammation in about 80% of refractory uveitis with relatively few serious adverse reactions.[23–25] However, repeated infusions every four to eight weeks are often required to prevent recurrences. In BD, the response to infliximab is especially rapid, occurring as early as 24 h after the infusion, even in patients who have recurrences despite being heavily immunosuppressed.[26,27] Adalimumab is given as a subcutaneous injection of 40 mg at weekly to two-weekly intervals; hence it can be self-administered, and because it is fully humanized, there is less likelihood of formation of antibodies. There has been only one report on its use in three adult patients with BD and three small studies in childhood uveitis. All three Behcet's patients had achieved remission with infliximab but were switched to adalimumab as it could be self-administered.[28] Etanercept is given twice weekly also as a subcutaneous injection of 25 mg and is consistently found to be less effective than the other two agents in uveitis. This is attributed to its relatively weak binding, mainly to the transmembrane form of TNF-α.[23,24] One potentially fatal complication of anti-TNF-α therapy is disseminated tuberculosis. Screening for latent tuberculosis may be compromised by the fact that these patients are usually already on other immunosuppressants, which may result in a false-negative purified protein derivative test, and the primary focus may be extrapulmonary.

### Cytokine receptor antibodies

Daclizumab is a humanized monoclonal antibody that targets the CD25 subunit of the human interleukin-2 receptor of T lymphocytes. It is given as an intravenous infusion of 1 mg/kg at two to four-week intervals. A subcutaneous form is currently still undergoing trials but shows promise as a more accessible route of administration.[29] Daclizumab has been found to be clinically beneficial in controlling the inflammation and, hence, preserving vision in birdshot chorioretinopathy, but is yet to be proved to be efficacious in the treatment of BD.

### Interferon-α2a

IFN-α2a is a cytokine released in viral infections and has been used mainly for the treatment of BD as it is thought to have a possible viral origin. It is given as a subcutaneous injection at a dose of 3–9 million units/day daily or thrice a week.[22,30]

Biologic agents are effective and comparatively well-tolerated options in the treatment of refractory uveitis in both adults and children in the short term, except for etanercept. The predominant side-effects of antimetabolites are bone marrow suppression and hepatotoxicity; that of T cell inhibitors is renal toxicity and that of alkylating agents are bone marrow suppression and the development of malignancies. The uncertainty of their long-term results, their high costs as well as the necessity for repeated intravenous infusions in the case of infliximab limit their widespread use.[31] Mycophenolate mofetil is another efficacious, fairly well-tolerated and less costly immunosuppressant. It has the additional advantage of an oral formulation.

#### Vitrectomy in panuveitis

Vitrectomy for uveitis began in the late 1970s for diagnostic purposes and for treating infections. Diagnostic vitrectomy combined with PCR can significantly improve diagnostic yield in otherwise idiopathic uveitis, and can frequently make a diagnosis in cases complicated by media opacity or other features that make traditional exam-based diagnosis difficult or impossible. Vitrectomy may be considered as a therapeutic option when uveitis persists despite maximum tolerable medical treatment with corticosteroids and/or other immunosuppressants. It may also be indicated when visual loss occurs due to complications of longstanding inflammations, such as a densely opacified vitreous, scar tissue pulling on the ciliary body causing hypotony, cystoid macular edema, an epiretinal membrane, a dense posterior lens capsule opacification or a tractional retinal detachment.[32] Vitrectomy removes the lodged lymphocytes in the vitreous, inflammatory debris, immune complexes and autoantigens. It also increases the uveal penetration of anti-inflammatory cells.[33] Besides providing a better access for complete removal of the cataractous lens material along with posterior capsule, the combined approach of pars plana lensectomy and vitrectomy allows easy performance of intraocular maneuvers and prevents formation of cyclitic membrane.[34]

Complications of vitrectomy may be mild or severe and include bleeding, cataract, glaucoma, infection, retinal detachment or blindness.

Some of the common uveitis entities seen in our population causing panuveitis are discussed.

#### Tuberculosis

Tuberculosis (TB)-related uveitis is being increasingly reported from Southeast Asian, Western Pacific, and Eastern Mediterranean regions.[3,35,36] The true prevalence of tubercular uveitis remains a major concern, especially in TB-endemic areas and because of lack of definite diagnostic criteria. TB can affect any part of the eye and patients present with a spectrum of clinical signs. Although it may mimic other clinical entities, a positive tuberculin skin test, healed lesions on chest X-ray or associated systemic TB corroborates the diagnosis of presumed intraocular TB. Administration of anti-tubercular therapy (isoniazid 5 mg/kg/day, rifampicin 450 mg/day if body weight is < 50 kg and 600 mg/day if body weight is > 50 kg, ethambutol 15 mg/kg/day, and pyrazinamide 25 to 30 mg/kg/day initially for three to four months; thereafter, rifampicin and isoniazid are used for another nine to 14 months) in addition to the standard corticosteroids significantly reduces recurrences of uveitis in these patients.[37] In our experience, the most common presentation of tubercular uveitis is posterior uveitis, followed by anterior uveitis. Panuveitis and intermediate uveitis are less common. The recently introduced immune-based rapid blood tests [QuantiFERON-TB Gold test (QFT-G) and TSPOT (*TB* test)] seem to be a significant upgrade of the century-old tuberculin skin test for diagnosing latent TB infection. QFT-G assay measures the amount of interferon-gamma (IFN- γ) released by the patient's sensitized T-cells when his whole blood is incubated with two synthetic peptide antigens ESAT-6 and CFP-10. These antigens are present in *Mycobacterium tuberculosis* but not in the Bacilli-Calmette-Guerin (BCG) or atypical mycobacteria. The advantages of these tests over the routine tuberculin skin test are that these are not affected by the previous BCG vaccination or atypical mycobacteria, the results are available within 24 h without any need for a second visit to the hospital and they are free from any booster effect.[38,39] The major limitation of these tests is that they are expensive.

#### VKH syndrome

**Figure 1:**
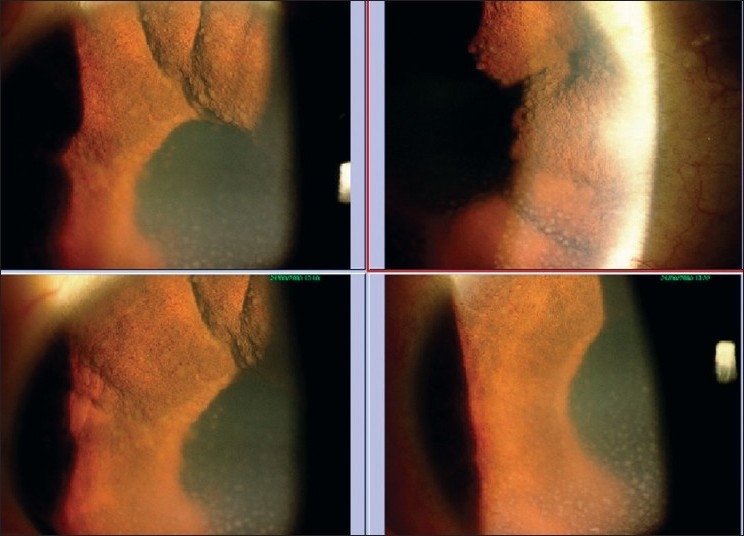
A 14-year-old girl having bilateral granulomatous panuveitis (left top and bottom showing the right eye, and right top and bottom showing the left eye)

VKH is a severe bilateral granulomatous panuveitis associated with various extraocular manifestations involving the central nervous, auditory, and integumentary system.[40] This syndrome usually affects adults between 20–50 years of age. Evidence suggests that it involves a T-lymphocyte–mediated autoimmune process directed against an as yet unidentified antigen (or antigens) associated with melanocytes.[41–43] The mechanism that triggers this autoimmune attack is unknown, but sensitization to melanocytic antigens by means of cutaneous injury, or viral infection have been proposed as possible factors in some cases. Although the exact target antigen has not been identified, candidates have been proposed. They include tyrosinase or tyrosinase-related proteins, an unidentified 75 kDa protein obtained from cultured human melanoma cells (G-361), and the S-100 protein. VKH disease is typically characterized as affecting primarily those of more pigmented groups, such as Hispanics (Mestizos), Asians, Native Americans, Middle Easterners, and Asian Indians, but not blacks of sub-Saharan African descent. This finding, in combination with the evidence of an increased risk among those with certain HLA genotypes, points to a genetically determined susceptibility to the triggering event for VKH disease.[44–49] Once diagnosed, multiple therapeutic regimens have been used in the treatment of VKH disease, including regional, oral, and intravenous corticosteroids, cyclosporine, antimetabolites, and alkylating agents. The diagnosis of VKH is clinical and several criteria have been proposed in the past, such as the American Uveitis Society (AUS) criteria[50] and Sugiura's criteria.[51] The revised diagnostic criteria subdivide patients into complete, incomplete, or probable categories according to the presence of extraocular manifestations.[52] VKH disease is a single entity with very different clinical manifestations depending on the stage at which the patient is examined. Thus, patients presenting soon after the onset of the disease may complain of auditory and neurological manifestations (severe headache and meningismus) followed by the onset of decreased vision, with the finding of exudative retinal detachments and optic disc hyperemia. Conversely, a patient presenting months to years after the initial episode will more typically have the signs and symptoms of an anterior uveitis with photophobia and cell and flare in the aqueous humor [Figure 1], along with the possible additional manifestations of cutaneous and ocular hypopigmentation. The ocular manifestations are divided into those occurring early and those occurring late in the course of the disease. In its early stages, there is the occurrence of a diffuse choroiditis manifested by a number of findings, including diffuse choroidal inflammatory signs and exudative detachment of the retina, the latter of which may appear as focal areas of subretinal fluid accumulation, development of larger bullous detachments, or both. When the patient presents months to years after the initial episode, he must demonstrate any of the late findings such as sunset glow fundus or Sugiura sign (perilimbal vitiligo) with nummular chorioretinal depigmentation scars (also sometimes erroneously referred to as Dalen-Fuchs nodules), generalized RPE clumping or migration, and recurrent or chronic anterior uveitis. The disease is typically characterized by exacerbations and remissions. The clinical course is variable: some patients may have limited inflammatory activity; while others have recurrent episodes of severe intraocular inflammation causing rapid visual loss.[53] VKH disease is a multisystem disorder. Unequivocal diagnosis therefore requires evidence of the involvement of more than the ocular system. When the patient presents at disease onset, evidence of meningeal inflammation in the form of malaise, fever, headache, nausea, abdominal pain, stiffness of the neck and back, or a combination of these features (commonly referred to as meningismus), or auditory involvement in the form of tinnitus is required. If neither meningismus nor tinnitus is present, pleocytosis on cerebrospinal fluid examination is required. Cutaneous findings such as vitiligo, poliosis or alopecia are late manifestations of this disease.

Apart from routine fundus photography and FA, ICGA has an immense role in diagnosing and following VKH patients. The major ICGA signs described in an acute initial uveitis episode include (1) early choroidal stromal vessel hyperfluorescence and leakage, (2) hypofluorescent dark dots, (3) fuzzy vascular pattern of large stromal vessels and (4) disc hyperfluorescence.[54] In the absence of an ICGA follow-up, undetected smoldering subclinical disease may persist. So VKH disease should be followed by ICG angiography and, in the case of choroidal subclinical reactivation, a reversal of therapy tapering and an extension of therapy duration should be considered.

**Figure 2:**
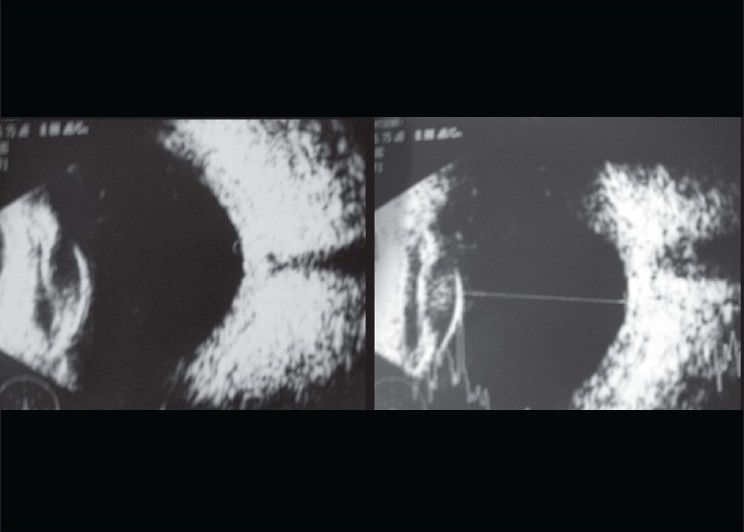
Ultrasound B-scan showed bilateral retinochoroidal thickening (arrows). Diagnosed as probable Vogt-Koyanagi-Harada's syndrome, she was treated with intensive topical betamethasone and mydriatics drops. She also received intravenous methyl prednisolone 1 g for five days followed by oral steroids

Traditionally, VKH disease has been treated with high-dose corticosteroids, often with immunosuppressive drug therapy reserved for patients with disease that is refractory to corticosteroid therapy. Treatment for VKH disease often is aimed at treating individual exacerbations of inflammation.. The typical treatment for VKH disease is high-dose corticosteroid therapy followed by a slow tapering of the drug over three to six months. The course of treatment of uveitis in such patients is usually aggressive, increasing the chances of sight-threatening complications like cataract, glaucoma and choroidal neovascular membrane. Our initial approach to the treatment of VKH-associated panuveitis includes frequent topical and systemic corticosteroids (1 mg/kg/day) along with cycloplegics [Figure 2]. Acute exacerbations require admission of the patient in the hospital and are controlled by intravenous methyl prednisolone injection (1 g/day) for three consecutive days followed by oral prednisolone (1.0-1.5 mg/kg/day). Immunosuppressive agents (azathioprine 2.0-2.5 mg/kg/day) are added as and when indicated. They are also started when a prolonged period of quiescence of inflammation is desired in the eye before undergoing an intraocular surgery (cataract, glaucoma, vitrectomy).

**Figure 3:**
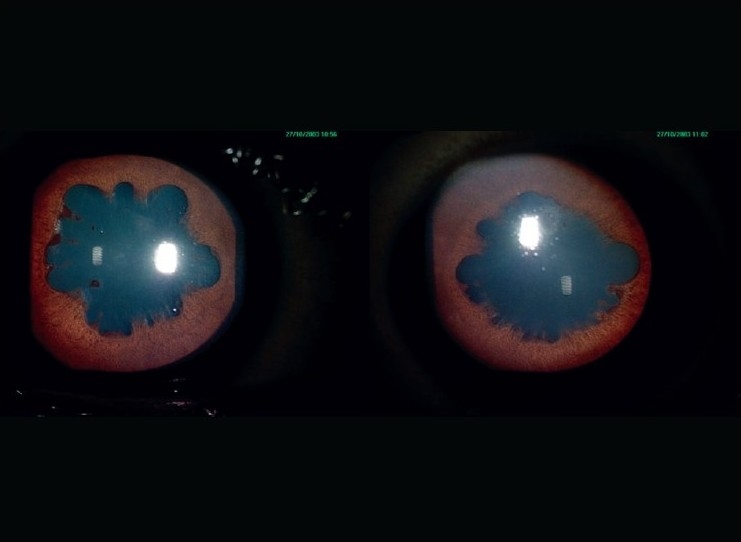
Anterior segment photographs of the right eye (on left) and left eye (on right) after three months of treatment; on maintenance dose of oral steroids 5 mg daily

**Figure 4:**
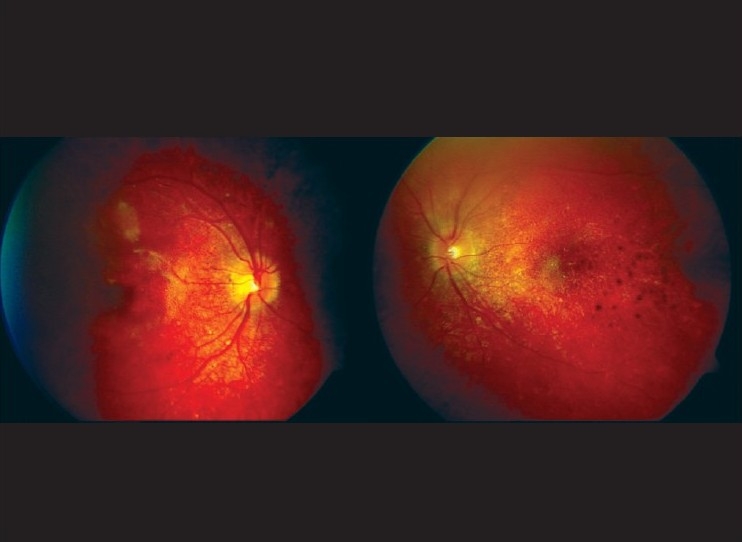
At three months, both eyes showing quiescent and sunset glow fundus

Once the inflammation is adequately controlled, the corticosteroids are tapered very gradually over months and years [Figures 3 and 4]. The patients are followed on low-dose topical and systemic corticosteroid with or without immunosuppressive agent as the maintenance therapy. Low-dose azathioprine therapy has been found to be effective as corticosteroid-sparing agent in VKH disease.[55]

Although relatively a rare cause of uveitis in children, VKH has been reported in children below 16 years of age during the last 50 years.[54,56–63] The course of VKH-associated uveitis is more aggressive in children than adults. Early use of corticosteroid-sparing agents has been recommended in pediatric patients because of hazards of cataract, glaucoma and growth retardation with long-term and high-dose corticosteroids.[64]

#### Sympathetic Ophthalmia

SO is an autoimmune condition in which injury to one eye (exciting eye) causes sight-threatening inflammation in the otherwise normal contralateral eye (sympathizing eye).[65] It typically presents as bilateral granulomatous panuveitis. It is a rare disease with an incidence of 0.03/1,00,000.[66] The classical description of signs include granulomatous mutton fat keratic precipitates, anterior chamber, and vitreous inflammation with or without yellow -white lesions in the retinal periphery. Other fundus lesions like retinal detachment, papillitis, optic atrophy, and vasculitis are reported uncommonly and are generally seen in conjunction with anterior segment inflammation.[67,68] There are no definite tests for confirming the diagnosis of SO. A history of an intraocular surgery or an ocular trauma combined with the clinical signs of inflammation support the diagnosis of SO. However, routine laboratory and ancillary tests are performed in the clinical practice to rule out the possibility of other granulomatous panuveitis conditions that mimic SO such as VKH syndrome or sarcoidosis. The mainstay of treatment is aggressive use of corticosteroids. All patients seen in the acute phase of disease in our clinic receive systemic corticosteroids with a starting dose of 1.5–2 mg/kg body weight that is tapered slowly to a maintenance dose between 5 and 10 mg/day over the next four to six months, which is then continued for a period of two to five years.[69] In case of recurrences, the dose of oral corticosteroids is increased to 1.5–2 mg/kg body weight if the patient has new fundus lesions or severe anterior segment inflammation. The maintenance dose, this time, is not reduced below the level at which the recurrence has occurred. Topical corticosteroids and cycloplegics are given as per requirement. Severe or recurrent cases require additional therapy, including more than one immunosuppressive agent. Patients seen in the chronic recurrent form receive oral azathioprine in a dosage of 100–150 mg/day to begin with, which is gradually reduced over the next 6–12 months. Patients who initially respond to but are unable to continue corticosteroids due to either side-effects (five patients) or recurrence (three patients) also receive immunosuppressive therapy. Intravenous cyclophosphamide (750 mg/ week every three to four weeks with a maximum of seven doses) is given for managing acute recurrences while oral azathioprine (100–150 mg/day) or methotrexate (7.5–10.0 mg/day) is used as steroid sparer.

#### Behcet's disease

BD is a multisystem disorder characterized by recurrent eye inflammation, oral ulcers and genital ulcers. Eye involvement, which affects 60–80% of BD patients, is characterized by unilateral or bilateral acute episodes of iridocyclitis with or without hypopyon, and/or panuveitis.[70–72] The majority of patients with ocular BD present with recurrent panuveitis.[73] There is evidence that the disease is more severe with the risk of losing useful vision higher in men than in women.[74] Eye disease in BD patients is mostly a recurrent nongranulomatous uveitis with necrotizing obliterative retinal vasculitis, which may be found either in the anterior or the posterior segment, or both.[75–78] It affects the posterior eye segment more often and more severely than the anterior one. Diagnosis of BD is mostly based on several sets of diagnostic criteria. Today the most widely used are the criteria of the international study group for BD.[79] However, these were developed as classification and not as diagnostic criteria, so, especially in early stages of the disease, the diagnosis of BD is often very difficult.[80] Older sets of criteria, most commonly those by Mason and Barnes,[81] O'Duffy[82] and Dilsen *et al.*,[83] and in Asia the Japanese,[84] are therefore still in use.

Systemic corticosteroids are widely used in the therapy of ocular BD. They are usually administered as oral prednisolone at an initial dose of 1–2 mg/kg/day, followed by a gradual tapering by 5–10 mg/week. However, they are not always suitable as a monotherapy for maintaining remission of uveitis due to adverse side-effects. In such cases, it becomes necessary to add an immunosuppressive drug as a steroid-sparing agent.

Cyclosporine A is the only immunosuppressant approved for uveitis therapy in several countries and is known to be the most commonly used immunosuppressive drug for ocular BD. Along with low-dose corticosteroids, it has been proved to be effective and safe for treating acute uveitis episodes as well as for reducing recurrence rates of uveitis in ocular BD at a dose of 3–5 mg/kg/day.[85–87] Azathioprine, in a dose of 2.5 mg/kg/day has also been shown to effectively control intraocular inflammation, to maintain visual acuity, and to prevent onset or progression of eye disease in ocular BD.[88]

Despite aggressive immunosuppressive treatment, the visual prognosis of ocular BD remains generally poor. Open-label clinical trials in Japan and Turkey have shown infliximab to be effective in improving the prognosis of the disease in ocular BD.[89,90] IFN-α has antiviral, antiproliferative and various immunomodulatory effects.[91] Unfortunately, these new drugs are very expensive and therefore they may be not universally available in countries with a low economic status.

The treatment of BD has to follow a multidisciplinary approach because of possible involvement of multiple organs, which necessitates early referral of patients to specialized internists with experience in diagnosis and treatment of this disorder. The fact that no standardized treatment regimens exist complicates the treatment of ocular BD.

#### Sarcoidosis

The organs affected more often are the lungs, skin, and eyes. The frequency of ocular involvement ranges from 26-50%.[92] Anterior segment involvement has been reported to be the most common. Panuveitis occurs in 6-33% of patients with sarcoidosis.[93–96] Presence of panuveitis is considered as a poor prognostic factor in patients with sarcoidosis.[93,97,98] The gold standard in the diagnosis of sarcoidosis is histopathological evidence of noncaseating granuloma.[99] The routine clinical tests for diagnosing sarcoid uveitis include Mantoux test, chest X-ray, serum angiotensin converting enzyme levels, erythrocyte sedimentation rate. When suspecting extraocular sarcoidosis, biopsy of the involved organ is performed to confirm the diagnosis. Otherwise, in all suspected cases of sarcoid uveitis, the diagnosis remains presumptive. A negative Mantoux test that corroborates the clinical diagnosis of sarcoidosis can be explained by a preponderance of suppressor cells in the peripheral blood leading to depletion of T-helper cells and monocytes that actually cause the delayed hypersensitivity response.

Sarcoid uveitis is uncommon in the Asian Indian population and its diagnosis often remains clinical.[3,5,6] In a series of histologically confirmed systemic sarcoidosis with ocular involvement in India, anterior and intermediate uveitis have been found to be the commonest sites.[92,100]

Besides topical corticosteroid and cycloplegic eye drops for anterior uveitis, systemic corticosteroids (prednisolone 1 mg/kg/day) are the mainstay of treatment for intermediate, posterior or panuveitis. Unilateral cases may benefit from posterior subtenon injection of triamcinolone acetonide (20 mg). The oral corticosteroids are tapered over 8-10 weeks by 5-10 mg per week depending upon the clinical response, in consultation with the pulmonologist. Although immunosuppressive agents in general have had very limited use in the treatment of ocular sarcoidosis, a state of controlled inflammation has been achieved and reported by a few authors using low-dose Methotrexate in the otherwise difficult-to-treat patients of panuveitis.[19,101] Beneficial effect of Methotrexate in patients undergoing cataract surgery has also been observed by a few authors in controlling the preoperative inflammation (that was otherwise persistent on corticosteroids alone) and sustaining improved visual acuity after surgery.[19] Because sarcoidosis is a multisystem disorder, the immunosuppressive drugs also help in managing the non-ocular manifestations of the disease. Certain side-effects may be seen with low-dose Methotrexate but they are better tolerated than those with long-term corticosteroids.[102]

## Conclusion

A systematic tailored approach in making accurate diagnosis is central to employment of specific, more effective treatment for all types of uveitis, irrespective of the anatomical site of inflammation.[1] What we prefer not to do is the exhaustive approach.

Uveitis will continue to be increasingly more important as a group of potentially sight-threatening inflammatory eye diseases that have a significant impact on both the visual and systemic health of the generally young adult population that is affected.[5] The use of a prospective analysis in a collaborative, multi-center approach would facilitate therapeutic trials and provide valuable breakthrough in managing some of the most refractory sight-threatening types of panuveitis. Therapeutic decisions are dictated by disease location and severity.

As uveitis often afflicts the young adult population in their most productive years of life, the personal and population burden of this sight-threatening disease is significant.[5] Irrespective of the cause, visual morbidity is poor in panuveitis. There is an increased risk of development of cataract, secondary glaucoma and cystoid macular edema as a result of more widespread inflammation and more aggressive treatment with corticosteroids. While identifying the causes of blindness in patients with intraocular inflammation, Rothova *et al*. found that panuveitis had the worst visual outcome as far as the anatomical site of involvement was concerned.[98]

The aim to treat a patient of panuveitis is to achieve a successful outcome in terms of quiescence of inflammation in both anterior as well as posterior segments, prevent recurrences of inflammation, minimal or no side-effects of chronic treatment which these patients receive, and favorable visual recovery in the long term. Although uveitis workup is teamwork requiring an accurate description of uveitis type and etiology, a selective approach to general investigations and an internist to monitor systemic treatment, an ophthalmologist plays a crucial role in the diagnosis and management of these patients.
